# Factors effecting influenza vaccination uptake among health care workers: a multi-center cross-sectional study

**DOI:** 10.1186/s12879-016-1528-9

**Published:** 2016-05-04

**Authors:** Süheyl Asma, Hülya Akan, Yücel Uysal, A. Gürhan Poçan, Mustafa Haki Sucaklı, Erhan Yengil, Çiğdem Gereklioğlu, Aslı Korur, İbrahim Başhan, A. Ferit Erdogan, A. Kürşat Özşahin, Altuğ Kut

**Affiliations:** Department of Family Medicine, Başkent University Faculty of Medicine, Bağlıca Kampüsü Eskişehir Yolu 20. km Bağlıca, Ankara, 06810 Turkey; Department of Family Medicine, Yeditepe University Faculty of Medicine, İnönü Mahallesi, Kayışdağı Cad., 26 Ağustos Yerleşimi, Kadıköy, İstanbul, 34755 Turkey; Department of Family Medicine, Mersin University Faculty of Medicine, Çiftlikköy Kampusu, Mersin, 33343 Turkey; Department of Family Medicine Sütçü İmam University Faculty of Medicine, Avşar Kampüsü, Kahramanmaraş, 46100 Turkey; Department of Family Medicine, Mustafa Kemal University Faculty of Medicine, Ürgen Paşa Mh, Hatay, 31030 Turkey

**Keywords:** Influenza, Vaccination behavior, Healthcare workers

## Abstract

**Background:**

The present study aimed to identify factors affecting vaccination against influenza among health professionals.

**Methods:**

We used a multi-centre cross-sectional design to conduct an online self-administered questionnaire with physicians and nurses at state and foundation university hospitals in the south-east of Turkey, between 1 January 2015 and 1 February 2015. The five participating hospitals provided staff email address lists filtered for physicians and nurses. The questionnaire comprised multiple choice questions covering demographic data, knowledge sources, and Likert-type items on factors affecting vaccination against influenza. The target response rate was 20 %.

**Results:**

In total, 642 (22 %) of 2870 health professionals (1220 physicians and 1650 nurses) responded to the questionnaire. Participants’ mean age was 29.6 ± 9.2 years (range 17–62 years); 177 (28.2 %) were physicians and 448 (71.3 %) were nurses. The rate of regular vaccination was 9.2 % (15.2 % for physicians and 8.2 % for nurses). Increasing age, longer work duration in health services, being male, being a physician, working in an internal medicine department, having a chronic disease, and living with a person over 65 years old significantly increased vaccination compliance (*p* < 0.05). We found differences between vaccine compliant and non-compliant groups for expected benefit from vaccination, social influences, and personal efficacy (*p* < 0.05). Univariate analysis showed differences between the groups in perceptions of personal risks, side effects, and efficacy of the vaccine (*p* < 0.05). Multivariate analysis found that important factors influencing vaccination behavior were work place, colleagues’ opinions, having a chronic disease, belief that vaccination was effective, and belief that flu can be prevented by natural ways.

**Conclusion:**

Numerous factors influence health professionals’ decisions about influenza vaccination. Strategies to increase the ratio of vaccination among physicians and nurses should consider all of these factors to increase the likelihood of success.

## Background

Influenza is a contagious disease associated with yearly seasonal outbreaks and significant mortality among risk groups [1]. During outbreaks, health professionals are repeatedly exposed to the influenza virus, and generally continue working even when infected. As the disease is often asymptomatic, health professionals can further spread the virus to their patients and families [2–4]. It has been demonstrated that the administration of influenza vaccine to health professionals is a cost-effective strategy that reduces lost work hours, as well as nosocomial transmission and mortality among hospitalized patients [5–8]. Previous studies have suggested that physicians who are vaccinated are more likely to recommend the influenza vaccine to their patients, and physician and nurse attitudes are important factors influencing patients’ decisions about vaccination [9, 10].

Similarly to Europe, Turkey has a low ratio of vaccination against influenza [11–13]. The targeted vaccination ratio was 45.5 % among health care workers (HCWs) in 2011, with a goal of increasing this ratio to 90 % by 2020 [14].

It is important to understand the attitudes and behaviors of health professionals toward vaccination to develop strategies to improve vaccination rates of health professionals and other individuals. There are a limited number of studies that have investigated the vaccination rates and vaccination status of health professionals, with data particularly limited in Turkey. The majority of the available studies are focused on specific areas or hospitals. Factors affecting decisions about getting vaccinated or not may change from country to country, especially in terms of social and organizational factors.

The present study aimed to investigate the attitudes and behaviors toward influenza vaccination, and factors influencing vaccination behavior of health professionals working in the south-eastern region of Turkey. Based on previous data, we focused our investigation on factors such as years worked, age, gender, sources of knowledge, health status, severity of perceived risks, perceived benefits, perceived barriers, motivating factors, attitudes, social effects, and personal efficacy.

## Methods

The present cross-sectional study was conducted between 1 January 2015 and 1 February 2015 using a self-report questionnaire.

The study population consisted of nurses and physicians who were working in university hospitals in the south-eastern region of Turkey, and who agreed to participate and gave informed consent. There are six university hospitals in the study region, and a total of 3650 health professionals (1551 physicians and 2099 nurses) were invited to participate. One university hospital did not participate, meaning five university hospitals (Baskent University Adana Hospital, Mersin University, Hatay Mustafa Kemal University, and Kahramanmaraş Sutcu Imam University) were enrolled in the study. This gave a sample of 2870 health professionals (1220 physicians and 1650 nurses). Staff email address lists were filtered for physicians and nurses by the five participating hospitals and provided to the present researchers. All health professionals were contacted via email and asked to complete the questionnaire electronically. Questionnaires were sent to participants three times during the study. The targeted minimum response rate was 20 %.

The questionnaire comprised two sections. The first section included multiple-choice questions concerning demographic characteristics such as age, gender, occupation, years worked in health services, and the institute and department in which the participant worked. Intensive care units, surgical areas and emergency medicine units accepted as “high risk areas”. Vaccination status has been asked as “Do you vaccinate against influenza?” The anwer choices were “I have never vaccinated”, “I have vaccinated before but I do not vaccinate every year” and “I regularly vaccinate every year”. HCWs answering “I regularly vaccinate” accepted “vaccine compliant” and others accepted “vaccine non-compliant”.

The second section focused on influenza vaccination, and covered behavioral factors assessed by five-point Likert-type questions of total 50 questions. The answers were expressed as: 1 is “I strongly agree”, 2 is “I agree”, 3 is “neutral”, 4 is “I disagree”, 5 is “I strongly disagree”. The questions were adapted from a previous study conducted among primary care health workers in 2015 (Akan et al., unpublished). In that study a questionnaire have been prepared based on studies and approach of Looijmans-Van den Akker et al. and Hopman et al. and relevant Turkish literature and recommendations from the Ministry of Health [15, 16]. The present researchers used a consensus-based approach to further adapt questionnaire items to secondary and tertiary health professionals, and the content of some items were changed. The primary domains of this section of the questionnaire were the severity of the perceived risks, perceived benefits, perceived barriers, motivating factors, attitudes, social effects, and personal efficacy. The Cronbach’s alpha coefficient for the questionnaire was calculated as 0.92 in a pilot study conducted with 35 physicians and nurses at Baskent University.

We asked participants about the status of regular vaccination to assess vaccination compliance. Participants who reported having been regularly vaccinated were considered as the vaccination compliant group, and those who had never been vaccinated or vaccinated only once were considered to be the vaccination non-compliant group.

### Statistical analysis

Categorical variables were summarized by number and percentage, and continuous variables were described by mean, standard deviation (SD), median, inter-quartile range, minimum, and maximum. The vaccination compliant and non-compliant groups were compared using Mann Whitney U tests due to a non-normal distribution pattern for continuous variables. Categorical variables were compared using chi-square or Fisher’s exact tests.

Estimating effects of independent variables on vaccination habits were evaluated with univariate logistic regression models. Variables had statistically significant estimating effect on vaccination habits (*p* < 0.10) were included to mutually adjusted multivariate logistic models and results of the model with the best Bayesian information criterion (BIC) value were reported. Regression results were summarized with odds ratio (OR), 95 % confidence interval (CI) boundaries of the OR, and *P* values.

The type 1 error level was set at 0.05, as the hypothesis was two-sided. Analyses were performed on SPSS software version 21.0 (IBM SPSS Statistics for Windows, Version 21.0. Armonk, NY: IBM Corp.).

We obtained ethics committee approval from Baskent University (project number: KA 15/08).

## Results

Responses were received from 642 to 2870 health professionals, giving a response rate of 22.4 %. In total, 628 subjects were included in the analysis; 14 participants were excluded as they did not respond to the question about vaccination habits. The mean age of the participants was 29.6 ± 9.2 years (range 17–62 years); 177 (28.2 %) were physicians and 448 (71.3 %) were nurses, 406 (64.6 %) were female and 218 (34.7 %) were male. Of the participants, 397 (63.28 %) were from Adana, 149 (23.7 %) were from Kahramanmaras, 47 (7.5 %) were from Mersin, and 34 (5.4 %) were from Hatay.

The ratio of participants who are vaccine compliant was 9.2 % (15.2 % of physicians and 8.2 % of nurses). When the ratio of the participants was evaluated with regard to departments, the highest vaccination ratio was found among internal medicine workers (53.4 %) followed by surgery departments (25.9 %), intensive care unit workers (10.3 %), emergency department workers (5.2 %) and others (1.7 %). The vaccination rate difference between health care professionals working in high-risk areas and low risk areas was statistically significant (*p* < 0.05) and was higher in people working in low risk areas.

### Univariate analysis

#### Univariate logistic regression analysis showed (Table 1, Table 2)

**Table 1:** Demographic characteristics and knowledge sources of health professionals

Participant opinions	Vaccination compliant (*n* = 58)^a^	Vaccination non-compliant (*n* = 570)^a^	*P*
Demographics			
Age (years)	36.6 (±9.8)	28.9 (±8.2)	<0.0001
Gender (male)	28/58 (48.3 %)	190/570 (33 %)	0.025
Working years	13.1 (±8.4)	6.5 (±6.5)	<0.0001
Influenza related risk factors			
Having a chronic disease	7/49 (14.2 %)	13/546 (2.4 %)	0.001
Living with a high risk person			
Living with a child aged <2 years	17/58 (29.3 %)	156/570 (27.4 %)	0.752
Living with a person with a chronic disease	22/58 (37.9 %)	231/570 (40.5 %)	0.701
Living with a person aged >65 years	22/58 (37.9 %)	122/570 (21.4 %)	0.004
Living with a pregnant woman	7/58 (12.1 %)	78/570 (13.7 %)	0.732
Current information sources			
Newspaper and television	25/58 (43.1 %)	320/570 (56.1 %)	0.057
Social media	17/58 (29.3 %)	267/570 (46.8 %)	0.011
Health institutes	29/58 (50 %)	347/570 (60.9 %)	0.107
Ministry of Health website	20/58 (34.5 %)	202/570 (35.4 %)	0.885
Health websites	17/58 (29.3 %)	188/570 (33 %)	0.570
WHO and CDC websites	24/58 (41.4 %)	112/570 (19.6 %)	0.0001
Flu platform	7/57 (12.1 %)	48/570 (8.4 %)	0.349
Colleagues	27/58 (46.6 %)	287/570 (50.4 %)	0.581
No information received	1/58 (1.7 %)	53/570 (9.3 %)	0.049
Preferred information source			
Newspaper and television	21/58 (36.2 %)	231/570 (40.5 %)	0.523
Social media	14/58 (24.1 %)	206/570 (36.1 %)	0.068
Health institutes	35/58 (60.3 %)	367/570 (64.4 %)	0.541
Ministry of Health website	27/58 (46.6 %)	300/570 (52.6 %)	0.337
Health websites	19/58 (32.8 %)	186/570 (32.6 %)	0.984
WHO and CDC websites	26/58 (44.8 %)	197/570 (34.6 %)	0.120
Via e-mail	23/58 (39.7 %)	194/570 (34 %)	0.391
Flu platform	14/58 (24.1 %)	115/570 (20.2 %)	0.477
Via mail to personal address	13/58 (22.4 %)	120/570 (21.1 %)	0.830

*p value for comparisons

^a^Number of participants strongly agreeing or agreeing / total number of responses (percentage strongly agreeing or agreeing)

**Table 2:** Univariate analysis: behavioral determinants associated with influenza vaccine uptake among health professionals

Participant opinions	Vaccine compliant (*n* = 58)^a^	Vaccine non-compliant (*n* = 570)^a^	Odds ratio (95 % CI)	*P*
Perceived risk				
I have high risk for influenza	53/56 (94.6 %)	409/568 (72 %)	6.87 (2.12–22.30)	0.001
I can spread infection to my patients even if I am asymptomatic	46/56 (82.1 %)	352/566 (62.2 %)	2.80 (1.38–5.66)	0.004
Health professionals are under the highest risk in case of an epidemic	54/56 (96.4 %)	512/567 (90.3 %)	2.90 (0.69–12.22)	0.147
I can spread infection to my family even if I am asymptomatic	39/56 (69.6 %)	325/564 (57.6 %)	1.69 (0.93–3.05)	0.084
Severity of the perceived risk				
Influenza is dangerous for me	46/57 (80.7 %)	388/561 (69.2 %)	1.86 (0.94–3.69)	0.073
Influenza is dangerous for my patients	50/56 (89.3 %)	501/559 (89.6 %)	0.96 (0.40–2.35)	0.937
Influenza is dangerous for my family	52/57 (91.2 %)	478/564 (84.8 %)	1.87 (0.73–4.82)	0.194
Perceived benefit				
Vaccination reduces my personal risk	56/57 (98.2 %)	367/565 (65 %)	30.21 (4.15–219.90)	0.001
Vaccination reduces the risk of spreading the disease to my patients	53/55 (96.4 %)	373/561 (66.5 %)	13.36 (3.22–55.40)	<0.001
Vaccination reduces the risk of spreading the disease to my family	53/56 (94.6 %)	372/568 (65.5 %)	9.31 (2.87–30.17)	<0.001
Community vaccination reduces my workload during an epidemic	52/55 (94.5 %)	382/562 (68.0 %)	8.17 (2.52–26.51)	<0.001
Perceived barriers				
I don’t expect a side effect after vaccination	29/56 (51.8 %)	183/563 (32.5 %)	2.23 (1.28–3.88)	0.004
The inactive influenza vaccine currently available in our country is effective	46/56 (82.1 %)	204/557 (36.6 %)	7.96 (3.93–16.11)	<0.001
Allergic reaction against influenza vaccine is rare, or none	42/57 (73.7 %)	190/561 (33.9 %)	5.47 (2.96–10.11)	<0.001
Autoimmune disease development risk is rare, or none, after influenza vaccine	40/57 (70.2 %)	189/566 (33.4 %)	4.69 (2.59–8.50)	<0.001
I am not against vaccination	51/56 (91.1 %)	433/562 (77.0 %)	3.04 (1.19–7.77)	0.020
Need for vaccination every year negatively effects my regular vaccination	30/54 (55.6 %)	237/549 (43.2 %)	1.65 (0.94–2.89)	0.083
One can catch influenza even if vaccinated	49/55 (89.1 %)	451/557 (81.0 %)	1.92 (0.80–4.60)	0.144
I had side effects from my previous influenza vaccinations	21/57 (36.8 %)	133/553 (24.1 %)	1.84 (1.04–3.27)	0.036
The influenza vaccine itself does not cause influenza	26/56 (46.4 %)	215/557 (38.6 %)	1.38 (0.79–2.39)	0.254
Health professionals should be vaccinated even if patients have been vaccinated	50/56 (89.3 %)	327/562 (58.2 %)	5.99 (2.53–14.20)	<0.001
I find injection every year uncomfortable	27/55 (49.1 %)	285/563 (50.6 %)	0.94 (0.54–1.64)	0.828
Vaccination does not reduce the overall immunization	39/56 (69.6 %)	330/564 (58.5 %)	1.63 (0.90–2.95)	0.108
I believe the vaccines are useful	52/55 (94.5 %)	432/562 (76.9 %)	5.22 (1.60–16.98)	0.006
I believe in alternative medicine	40/56 (71.4 %)	396/561 (70.6 %)	1.04 (0.57–1.91)	0.895
I believe that natural methods are better than vaccination	23/55 (41.8 %)	379/562 (67.4 %)	0.35 (0.20–0.61)	<0.001
I am against vaccination due to my beliefs	6/54 (11.1 %)	111/563 (19.7 %)	0.51 (0.21–1.22)	0.130
Motivating factors				
I know the Ministry of Health recommendations about influenza vaccination	45/55 (81.8 %)	359/561 (64.0 %)	2.53 (1.25–5.13)	0.010
I know the Ministry of Health recommendations about the age groups and chronic diseases which require influenza vaccination	44/55 (80.0 %)	318/564 (56.4 %)	3.09 (1.57–6.12)	0.001
I have sufficient knowledge about influenza	47/55 (85.5 %)	408/561 (72.7 %)	2.2 (1.02–4.77)	0.045
I get knowledge about influenza from reliable sources every year	43/56 (76.8 %)	310/561 (55.3 %)	2.68 (1.41–5.09)	0.003
The Ministry of Health provides free vaccination for health professionals	41/55 (74.5 %)	310/561 (55.3 %)	2.37 (1.26–4.45)	0.007
Attitudes				
I feel that health professionals’ not spreading the disease to their patients important	53/57 (93.0 %)	480/562 (85.4 %)	2.26 (0.80–6.42)	0.125
I believe that health professionals should be vaccinated for the continuity of health services	51/56 (91.1 %)	363/557 (65.2 %)	5.45 (2.14–13.88)	<0.001
Right of choice for vaccination should be preserved for health professionals	43/57 (75.4 %)	480/557 (86.2 %)	0.49 (0.26–0.94)	0.033
Influenza vaccine should be mandatory for health professionals	36/56 (64.3 %)	193/557 (34.6 %)	3.39 (1.91–6.03)	<0.001
Social effects				
My relatives believe that my vaccination is important	48/57 (84.2 %)	252/560 (45 %)	6.52 (3.14–13.54)	<0.001
My institute recommends my vaccination	46/57 (80.7 %)	284/560 (50.7 %)	4.06 (2.06–8.01)	<0.001
My colleagues believe that my vaccination is important	47/56 (83.9 %)	250/558 (44.8 %)	6.43 (3.09–13.38)	<0.001
The Ministry of Health recommends vaccination of health professionals	44/55 (80.0 %)	325/554 (58.7 %)	2.82 (1.43–5.57)	0.003
The health authorities I respect recommend vaccination	46/55 (83.6 %)	292/553 (52.8 %)	4.57 (2.19–9.51)	<0.001
Personal competence				
I would be vaccinated every year if I have enough time	48/53 (90.6 %)	194/373 (52.0 %)	8.86 (3.45–22.75)	<0.001
I would be vaccinated if someone reminds me	49/57 (86.0 %)	194/555 (35.0 %)	11.4 (5.29–24.55)	<0.001
I would be vaccinated every year if the vaccine is provided in my institute	50/55 (90.9 %)	208/557 (37.3 %)	16.78 (6.59–42.75)	<0.001
I would be vaccinated every year if I am rewarded	29/56 (51.8 %)	144/558 (25.8 %)	3.09 (1.77–5.39)	<0.001
I would be vaccinated every year if sufficient knowledge was given	47/56 (83.9 %)	253/555 (45.6 %)	6.23 (3.00–12.97)	<0.001

*CI* 95 % confidence interval of odds ratio, *P p* value for odds ratio

^a^Number of participants strongly agreeing or agreeing/total number of responses (percentage strongly agreeing or agreeing)

The median age (35.5 years) of the group who were vaccine compliant was significantly higher than the median age (26.0 years) of those vaccine noncompliant (*p* < 0.0001). The proportion of females (51.7 %) in the compliant group was significantly lower than that (66.0 %) in the non-compliant group (*p* = 0.025). The proportion of physicians (48.3 %) in the compliant group was significantly higher than that (26.1 %) in the non-compliant group (*p* = 0.0004). The median years worked in health services for the compliant group (13.1 years) was significantly higher than that (6.5 years) of the non-compliant group (*p* < 0.0001). In the compliant group, the percentage of respondents with a chronic disease who required vaccination (12.1 %) was significantly higher than that (2.3 %) in the non-compliant group (*p* = 0.001). The proportion of participants living with a person older than 65 years (37.9 %) in the compliant group was significantly higher than that (21.4 %) in the non-compliant group (*p* = 0.004).

#### Multivariate logistic regression analysis

As there were a small number of health professionals who were regularly vaccinated each year and all factors could not be included in the multiple regression analysis, we created a corrected multiple logistic regression model with the variables chosen through preliminary methods. Variables with *p* < 0.10 were included in the main multivariate regression analysis. So at the end, variables “the institution in which the participant worked”, “presence of a chronic disease that required influenza vaccine”, “colleagues think vaccination is important”, “the inactive influenza vaccine currently available in our country is effective”, “fighting influenza with natural methods is more effective than vaccination with regard to overall health” are included. Model has 91.3 % accuracy for likelihood of regular vaccination every year.

Working at Başkent University Adana Hospital, Kahramanmaraş Sütcü İmam University (reference: working at Mersin University) decreased the likelihood of regular vaccination every year by 0.25, 0.18, times respectively; estimating effect of working at Mustafa Kemal Universty was not statistivally significant. Having a chronic disease that required vaccination increased the likelihood of regular vaccination every year by 5.13 times. Strongly agreeing or agreeing that colleagues thought vaccination is important increased the likelihood of regular vaccination every year by 3.45 times. Strongly agreeing or agreeing that the inactive flu vaccine currently available in Turkey is effective increased the likelihood of regular vaccination every year by 6.31 times. Strongly agreeing or agreeing that protection with natural methods against flu is better than vaccination for overall health status decreased the likelihood of regular vaccination every year by 0.38 times.

## Discussion

This study has shown that increasing age, increasing working years, having chronic disease and living with a person over 65 years are important and positively affecting factors to be vaccinated against seasonal influenza. In all behavioral domains there are differences between vaccine compliant and non-compliant groups; nearly all sub-items of perceived risk, perceived benefit, social effects and personal competence differed between groups. Also, knowing the recommendations of MoH, the thoughts of collegues, getting knowledge from reliable sources and thinking that “natural methods are better than vaccine to fight against flu” seem important factors affecting HCWs vaccination behavior. There were also differences between hospitals.

Recommendations about indications for influenza vaccine and reimbursement for vaccination vary between countries, and vaccination of health professionals has a similar variance. While influenza vaccine is recommended for health professionals and is provided free of charge in some countries, in other countries, vaccination is mandatory for health professionals in certain specialties [17, 18]. In Turkey, risk groups for influenza vaccination were identified in 2004, and the Ministry of Health has provided the vaccine to health care workers free of charge since 2011 to motivate health professionals to get vaccinated.

With the exception of Romania, where vaccination rates are high, vaccination rates vary from 14 to 15 % in European countries and are under the targeted rates [11, 14, 19–22]. Rates of influenza vaccination are also low in the general population in Turkey [12, 13].

Although the present study was not conducted with a selected sample, vaccination rates were low among participants who were regularly vaccinated, particularly nurses. Another striking finding was lower vaccination rates among health professionals who have more contact with critically ill patients.

Although previous studies used different methods, factors influencing vaccination behavior common to previous research and our study were demographic factors (age, working years, and presence of a chronic disease), risk perception, expected benefit, incorrect information and attitudes about the vaccine, social factors, and organizational insufficiencies [15, 23–25]. Institutional sufficiency may be important since there are differences between institutes in our study. The institutions included in the study, HCWs are informed by e-mail regarding influenza vaccine at the beginning of the winter and provide information about vaccine form their official web-sites also. Also, in Baskent University a nurse responsible from monitoring influenza vaccination among HCWs, inform workplace physician and head of departments by telephone. Still, there are some differences between institutions regarding implementation of flu vaccine which may explain the differences in vaccine compliance regarding workplace.

In our study, while risk perception changed vaccination status, perceived risk severity was high in both the compliant and non-compliant groups and this did not lead to a difference in vaccination behavior. There were significant differences in all components of the expected benefits, and the perception of expected benefits was clearly low in the non-compliant group. Another interesting finding was that although health professionals reported that their knowledge about the vaccine was sufficient, a high proportion of participants believed that the vaccine itself could cause influenza and side effects. The influenza vaccine currently available in Turkey is a trivalent inactive vaccine that is not likely to lead to influenza. Side effects of the vaccine are rare and the common side effects are no different than for other vaccines. We found that incorrect information and attitudes influenced vaccination behavior despite an individual’s belief that they had sufficient knowledge.

Another important factor affecting vaccination behavior was protecting the individual’s family and environment. This is also part of professional life for healthcare workers. Although do good for others is a good motivator this reason alone was not always reflected in vaccination behavior; Individuals decide to get vaccinated if others can benefit from their vaccination if their personal risk of vaccination was low; not vaccinated if their risk is high. In addition, time concerns and costs also played important roles in vaccination decision-making [26, 27].

There are many factors that affect behavior of vaccination against influenza. Many strategies have been proposed to improve the vaccination rates of health professionals. The most effective strategy seems to be making vaccination mandatory which has been implemented in some countries as United States succesfully 2015 survey has shown that the highest vaccination coverage was reported among HCW with an employer requirement for vaccination [28]. However, this strategy is controversial with regard to ethical concerns. While those who advocate mandatory vaccination propose that this is part of physicians’ professional responsibility to do no harm, the opponents claim that physicians’ self-rule should be preserved [27–30]. Although mandatory vaccination increases vaccination rates, it is negatively perceived by health professionals and this view should be respected [31]. Interestingly in our study, although the compliant group expressed more support, even in non-compliant group 34.6 % of HCWs support mandatory vaccination. Interventional studies in the literature are limited, and the majority of available studies focus on evaluating information and education [32].

Although having sufficient knowledge about influenza and vaccination has been shown to be an important factor in addressing incorrect knowledge and beliefs about the vaccine, and the need for education has been emphasized, many studies have shown that sufficient knowledge alone was not enough to changing vaccination behavior [10, 26, 33–36]. One study showed that physicians’ analytic knowledge was no different from that of the general population when risk perception was low, and physicians’ attitudes were no different from the general population when risk perception was high; their behavior was affected by their experiences and feelings [37]. Other studies have also shown that physicians’ behaviors with regard to their own health are similar to those of the general population [26].

Single interventions such as providing information or providing vaccination free of charge have been found to not influence vaccination behavior [35, 36]. Strategies targeting multiple interventions that are based on a comprehensive assessment seem to be more successful [37–39].

Our study has some limitations. First, the ratio of participation in our study was relatively low. Second, the study was conducted in one region and included only physicians and nurses so the results cannot be assumed to represent all healthcare workers in Turkey. Third, there was a possibility of bias due to participants with favorable attitudes towards influenza vaccination potentially being more likely to have responded to the study questionnaire or vica versa. Fourth, omitting free-text response options about factors affecting vaccination behaviors may have resulted in factors that were not included in the questionnaire being missed. However, the main goal of the present study was to identify factors that influence the decisions of health professionals about influenza vaccination. In this context, we believe that the findings of the present study may serve as a guide for the development and implementation of national-level strategies intended to increase the ratio of vaccination.

## Conclusions

There are numerous factors that influence the decisions of health professionals regarding influenza vaccination. Strategies aimed to increase the ratio of vaccination among physicians and nurses that consider all of these factors are more likely to be successful. In the planning and implementation of strategies to increase the ratio of vaccination among health professionals, it is necessary and important to consider changeable factors relating to individual behavior, as well as organizational factors.

### Ethics and consent to participate

This study was approved by the Institutional Medical and Health Sciences Experimental/Clinical Research Principles and Research Committee (project number: KA15/08). The required approvals for the collaborations in the study were obtained from the concerned departments and hospitals. The participants were informed about the objcetive and the moethod of the study and they were accepted to consent if they filled out the questionnaire form.

### Consent to publish

Not applicable.

### Availability of data and materials

Identifying/confidential patient data should not be shared.

## Abbreviations

CI: confidence interval
HCWs: health care workers
OR: odds ratio
SD: standard deviation
